# MEK inhibition prevents human skin graft rejection by promoting CD8^+^TCF1^+^ over CD8 effector T cells

**DOI:** 10.1016/j.isci.2025.113310

**Published:** 2025-08-06

**Authors:** Christine Chauveau, Veronique Nerriere-Daguin, Maeva Fourny, Cynthia Fourgeux, Thibaut Larcher, Laurence Delbos, Martin Braud, Lucas Brusselle, Olivia Rousseau, Jeremie Poschmann, Julien Verdier, Fabienne Haspot, Gilles Blancho, Simon Ville

**Affiliations:** 1 CHU Nantes, Nantes Université, INSERM, Center for Research in Transplantation and Translational Immunology, UMR 1064, ITUN, 44000 Nantes, France; 2 Chirurgie Plastique Reconstructrice et Esthétique, CHU de Nantes, Nantes, France; 3 INRAE Oniris, PAnTher, APEX, Oniris, Nantes, France; 4 Institut de Transplantation Urologie Néphrologie (ITUN), Service de Néphrologie et Immunologie Clinique, CHU Nantes, Nantes, France

**Keywords:** Cell biology, Immune response, Transcriptomics

## Abstract

Pharmacological MEK inhibition might be an innovative approach to complete the immunosuppressive regimen that enables solid organ transplantation. While MEK inhibitors like trametinib are approved in oncology, their immunomodulatory properties remain poorly investigated in the context of organ transplantation, especially in human context. Using a human skin transplantation model in NSG mice reconstituted with third-party human PBMCs, we evaluated the effects of trametinib on graft survival and the human allogeneic immune response. MEK inhibition significantly prolonged graft survival without reducing graft infiltrate, while preserving the human epidermal tissue. Single-cell RNA sequencing of splenic cells revealed that MEK inhibition impaired CD8^+^ T cell differentiation into effector phenotypes, favoring an accumulation of CD8^+^ TCF1^+^ stem-like cells. Additionally, MEK inhibition supported CD4^+^ T cell homeostasis by maintaining IL-7R expression. These findings suggest that MEK inhibition may simultaneously control the alloimmune response and support immune recovery, highlighting its potential in solid organ transplantation.

## Highlights

• MEK inhibition delays rejection of human skin grafts in humanized mice
• It limits CD8^+^ effector T cells while promoting CD8^+^ TCF1+ stem-like cells
• It preserves CD4^+^ T cell homeostasis by maintaining IL-7R expression

## Graphical abstract

**Figure:**
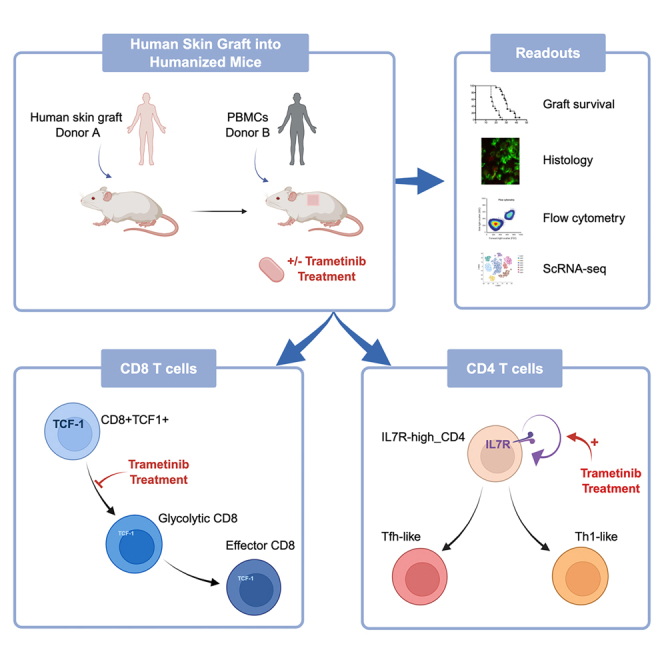

## Introduction

Solid organ transplantation remains the elective care for many end stage diseases. While the challenges vary among different organs, a common goal is to provide treatments that both prevent alloimmune-mediated damages of the transplant and are well tolerated. Indeed, while rejection still accounts for a large proportion of graft loss, the adverse effects of immunosuppressive regimens contribute to patient morbidity and mortality from infectious diseases, cancer, cardiovascular diseases, and direct toxicity.^1^

The RAS/mitogen-activated protein kinase (MEK)/extracellular signal-regulated kinase, i.e., MEK-ERK pathway, has been implicated in many cancers when aberrantly activated and is therefore a target for the development of antagonist drugs, such as the MEK inhibitor trametinib.^2^ This drug was approved ten years ago for treating adults with specific BRAF V600 mutated cancer cells, including a large proportion of melanoma or non-small cell lung cancers.^3^^,^^4^ Because this pathway is involved in the downstream signaling of many membrane receptors in different cell types, its inhibition can lead to many off-target effects. For instance, MEK inhibition has been shown in mouse models to protect the kidney from fibrosis and to inhibit calcineurin inhibitor-induced nephrotoxicity, two effects that could be beneficial in the setting of kidney transplantation.^5^^,^^6^

Although it has long been known that the MEK-ERK pathway is involved in T cell signaling,^7^ the immunological effects of MEK inhibitors have mostly been studied in the context of anti-tumor immunity, where potential interference with checkpoint inhibitors could be detrimental.^8^ In the setting of alloreactivity, *in vitro* studies have shown that MEK inhibition suppressed naive alloreactive T cells while sparing antiviral responses, and *in vivo* studies have shown its therapeutic potential in a murine model of graft-versus-host disease (GVHD), where MEK inhibition selectively suppressed GVHD without compromising graft-versus-tumor (GVT) effects.^9^^,^^10^ Finally, MEK inhibition has been shown to attenuate graft rejection following lung transplantation in rats and to prolong islet allograft survival in mice.^11^^,^^12^

To further investigate the impact and the immunological effects of MEK inhibitors on alloimmune-mediated damage in transplanted organs within a human context, we used a humanized preclinical model. This model consists of a human skin allograft in immunodeficient mice (NOD.Cg-Prkdcscid Il2rγtm1Wjl), subsequently reconstituted with allogeneic human PBMCs from a third donor, leading to skin graft rejection after immune recovery in the absence of treatment.

## Results

### MEK inhibition delays human skin graft rejection in humanized NSG mouse without hampering immune reconstitution

**Figure 1:**
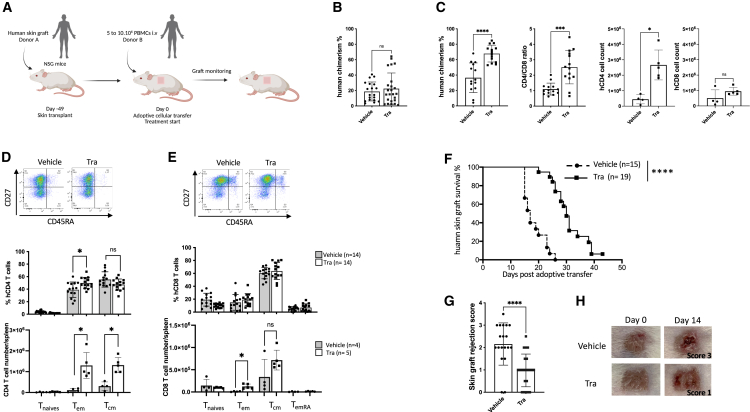
MEK inhibition delays allogenic human skin graft rejection without affecting immune reconstitution (A) NSG mice received a 1 cm^2^ split-thickness human skin graft followed by 5–10 × 10^6^ hPBMCs i.v (allogeneic to the skin) 7 weeks later. Trametinib (0.9 mg/kg) or vehicle was given daily by oral gavage. (B) Bar plot showing human chimerism assessed in the peripheral blood of mice at day 14. Vehicle treated group *n* = 15, trametinib treated group, *n* = 19. (C–E) Flow cytometry analysis of a replicated experiments on spleen harvested from animals at day 14 after hPBMCs injection, vehicle-treated group *n* = 9, trametinib-treated group *n* = 8. (C) Bar plots showing the human chimerism, the human CD4/CD8 ratio and the total number of CD4 and CD8 T cells. (D and E) Representative dot plot of flow cytometry analysis showing the T cell populations of naive T cells, (CD45RA^+^CD27^+^), central memory (CD45RA^−^CD27^+^), effector memory (CD45RA^−^CD27^−^) and TemRA (CD45RA^+^CD27^−^) T cells, along with bar plots showing the frequencies of each population and total spleen cell number for hCD4 (D) and hCD8 (E) T cell population in vehicle-treated mice (*n* = 10) and trametinib-treated mice (*n* = 9). (F and G) (F) Kaplan-Meier skin graft survival curve. Trametinib treatment delays allogeneic human skin rejection compared with vehicle-treated controls (30.7 ± 4.3 days, *n* = 17 vs. 18.4 ± 3.3 days, *n* = 15, log rank test ∗∗∗∗*p* < 0.0001. Pooled data of *n* = 3 experiments with skin from 3 different HD and hPBMC from 3 other different HD (G) Bar plot showing skin graft rejection scores at day 14 among vehicle-treated mice (*n* = 15) and trametinib-treated mice (*n* = 19). (H) Photographs representative of human skin grafts at day 0 or day 14 after allogeneic hPBMCs injection in vehicle and trametinib-treated animals. (B) and (D) are pooled data of *n* = 3 experiments with skin from 3 different HD and hPBMC from 3 other different HD. (C–E), and (G) are pooled data of *n* = 4 experiments with skin from 2 different HD and allogeneic hPBMC from 4 other different HD. (B–E), and (G) are shown as means ± SD, Mann-Whitney test. ns: non-significant, ∗*p* < 0.05, ∗∗∗*p* < 0.001, ∗∗∗∗*p* < 0.0001.

To investigate the effects of MEK inhibitors on graft rejection and immune reconstitution, we used a well-established model in which immunodeficient mice are first transplanted with a human skin patch, which is fully accepted, and subsequently infused with hPBMC from third-party human donor. Mice were orally daily treated with either trametinib (0.9 mg/kg) or vehicle from the day of hPBMC injection until the experiment end (Figure 1A). The trametinib dose was previously determined in NSG mice humanized by the injection of 5 to 10.10^6^ hPBMC to completely inhibit MEK phosphorylation (for which we observed a dose-response effect) (Figure S1) with no evidence of toxicity (weight evolution comparable to control mice). Human immune reconstitution was monitored weekly by analyzing human chimerism in peripheral blood and skin graft rejection was evaluated through daily blinded visual assessment. Importantly, the trametinib treatment did not impair human cell engraftment in mice bearing a human skin graft further humanized with hPBMC as we did not observe any significant difference in the human chimerism between the two groups’ blood samples (Figure 1B). All mice maintained consistent body weight (Figure S2), exhibited normal posture, and had a healthy fur appearance indicating the absence of xenogeneic graft-versus-host disease (GVHD). When analyzing the spleen at day 14, we observed a significant increase of human chimerism in the treated animals with a well-balanced CD4/CD8 ratio in the control group, unlike trametinib-treated animals which shifted toward CD4 T cells without affecting the proportion of naive (CD45RA^+^, CD27^+^), central memory (CD45RA^−^, CD27^+^) and effector memory (CD45RA^−^, CD27^−^) for both CD4 and CD8 T cell (Figures 1C–1E), (Figures S3 and S4). Interestingly, trametinib-treated mice displayed significantly prolonged human skin-graft survival with a mean survival of 30.7 ± 4.3 days compared to control mice at 18.4 ± 3 0.3 days (*p* < 0.0001) (Figures 1F–1H).

Thus, daily administration of trametinib, by inhibiting MEK phosphorylation, effectively delays the rejection of human skin grafts while preserving the establishment of human chimerism.

### MEK inhibition prevents skin epithelial cells injury beside immune cells graft infiltration

**Figure 2:**
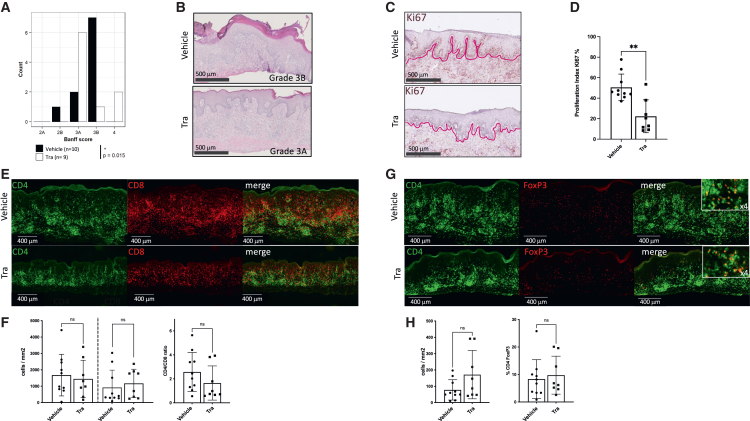
MEK inhibition limits the onset of human skin epithelial cell injury without affecting CD4 and CD8 allogeneic T cell infiltration Human skin grafts were harvested from transplanted NSG mice 14 days after the injection of allogeneic hPBMCs. Mice were daily treated with trametinib or vehicle from days 0–14. (A) Bar plot showing skin graft rejection blinded Banff score, using a validated grading scale, in vehicle- (*n* = 10) and trametinib-treated mice (*n* = 9), Fisher’s exact test ∗*p* < 0.05. (B) Representative H&E staining illustrating the difference between grade 3A (only single cell keratinocyte necrosis) and 3B (focal and full thickness necrosis). (C) Representative images of immunohistochemistry staining Ki67 (purple line delineates dermal/epidermal junction). (D) Bar plot showing the means ± SD of proliferation index of Ki67. (E) Representative images showing fluorescence patterns of hCD4 (green) and hCD8 (red) in human skin grafts of vehicle- and trametinib-treated mice. (F and G) (F) Bar plot showing the means ± SD of CD4 and CD8 human T cells (top) and the CD4/CD8 ratio (bottom) in the skin grafts (G) Representative images showing fluorescence patterns of CD4 (green) and FoxP3 (red) in human skin grafts. (H) Bar plot showing the means ± SD of human CD4 FoxP3 positive T cells (top) and their proportion among human CD4 T cells (bottom) in human skin grafts from vehicle and trametinib-treated mice. (D), (F), and (H) are pooled data of 4 experiments, vehicle-treated group *n* = 10, trametinib-treated group *n* = 8. Significance was calculated by non-parametric Mann-Whitney test. ∗∗*p* < 0.01.

To assess signs of skin rejection and inflammation at a specific time point, mice were euthanized 14 days post-adoptive transfer of allogeneic hPBMCs, which corresponds to a period approaching the onset of rejection in control animals. Histopathological evaluation of the grafts was subsequently conducted to compare the outcomes between the trametinib-treated and control groups. H&E-stained sections were analyzed blindly using a modified Banff grading system (Figure S5B). Most control animals exhibited focal and full-thickness epidermal necrosis (stage 3B) with infiltrating inflammatory cells in the dermal underlying tissue. In contrast, trametinib-treated animals displayed only multifocal single-cell keratinocyte necrosis (stage 3A) although the cellular infiltrate level was similar between the groups (Figures 2A and 2B). In the context of skin homeostasis, basal keratinocytes exhibit a constitutive level of Ki67 expression due to their proliferative role in maintaining epidermal renewal. Inflammatory skin damage observed during alloreactive immune infiltration induces hyperproliferation of basal keratinocytes resulting in an increased Ki67 index.^13^ Evaluation of the Ki67 positivity of the skin epidermal basal cells by immunohistochemistry showed that trametinib significantly reduced the Ki67 proliferation index confirming that the infiltrate appeared less harmful to epidermal cells in treated animals than in controls (Figures 2C and 2D). To investigate this discrepancy, we characterized the cell infiltrate by immunofluorescence staining and found no significant difference in density or composition for CD4 T cells, CD8 T cells and CD4 FoxP3^+^ T cells (Figures 2E–2H). Therefore, trametinib treatment protects human skin grafts despite the presence of both CD4 and CD8 lymphocytic infiltrates.

### Single-cell transcriptomic profile of trametinib-treated mice splenocytes

To elucidate how trametinib treatment affects anti-donor lymphocytic reactivity, we performed a comprehensive analysis using scRNA-seq, a method designed to capture the heterogeneity of a cellular population in an unsupervised way.

**Figure 3:**
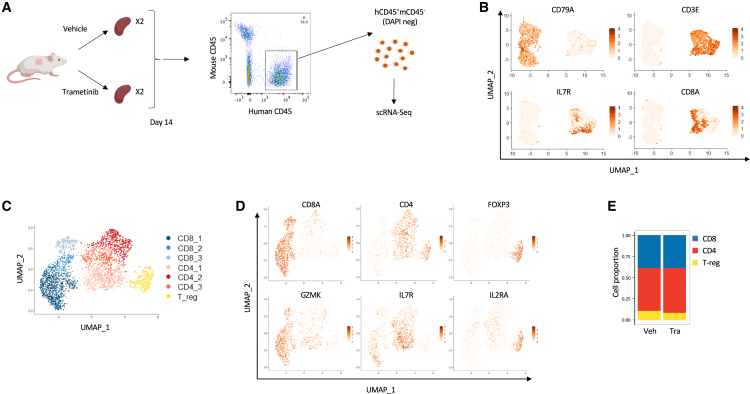
scRNA-seq cluster analysis of human CD45-positive cells isolated from the spleens of human skin grafted humanized mice (A) Mice treated with the vehicle or trametinib (two in each group) were sacrificed on day 14 post-reconstitution, living (negative DAPI staining) hCD45 positive spleen cells were sorted, labeled with oligo-tagged antibodies, pooled and subjected to 3′ single-cell RNA-seq on the 10× platform with the V(D)J sequencing. (B) UMAP projections based on reduction dimension using PCA applied to the entire dataset (after quality control) and colored by gene expression of B cell (*CD79A*) and T cell (*CD3E*, *IL7R*, *CD8A*) marker genes. (C) UMAP projections based on the reduction dimension after subsetting the dataset to the T cell subset, colored according to the result of clustering with the Louvain algorithm. (D) UMAP projections based on reduction dimension using PCA applied to the T cell subset and colored by gene expression of CD8 (*CD8A*, *GZMK*), CD4 (*CD4*, *IL7R*) and CD4 Treg (*FOXP3*, *IL2RA*) T cells marker genes. (E) Stacked barplot representing the proportion of each T cell subset (CD8, CD4 non-Treg, CD4 Treg) according to the treatment exposure (vehicle vs. trametinib).

We applied it to the spleens of two 14-day-treated mice in each group, all reconstituted with a unique third-party donor. Living human cells were sorted (Figure 3A), tagged with barcoding mAb prior to mixing and loading onto the chromium device, allowing them to be differentiated during analysis, eliminating any batch effect and minimizing potential doublets.

After quality controls (Figure S6) and doublets removal, we obtained 8861 cells with a mean number of 2228 ± 955 different expressed genes and 8074 ± 4634 counts, with 4277 cells from the control animals and 4584 from the trametinib-treated animals. We first differentiated T and B cell lineages based on *CD3E* and *CD79A* expressions, respectively (Figure 3B). We subsequently chose to focus on the 3452 T cells (1839 control and 1613 trametinib) due to the T cell nature of the graft infiltrate (Figure 3C). First, we visualized three groups that we identified as CD8, CD4, and CD4 regulatory T cells based on expression enrichment of *CD8A*/*GZMK*, *CD4*/*IL7R*, and *FOXP3*/*ILR2R*, respectively (Figure 3D). We did not observe any difference in their distribution between the treated animals and controls (Figure 3E), in line with the flow cytometry performed on the same experiment (Figure S7).

### Trametinib impairs CD8 differentiation into more potent effector cells

**Figure 4:**
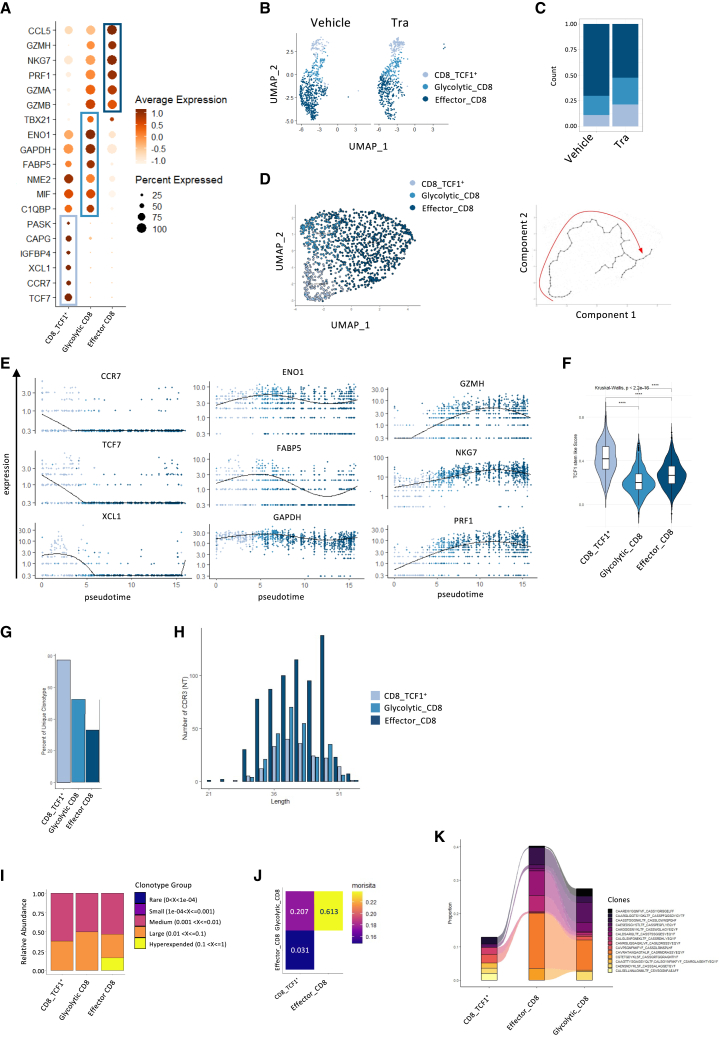
The CD8 compartment reveals a differentiation pathway from CD8^+^TCF1^+^ to effector CD8 cells impaired by trametinib (A) Dot plot depicting the expression profiles (average normalized expression and percentage) of genes identified as markers base on differential expression analysis, across the three CD8 clusters. (B) UMAP projection restricted to the CD8 subset, colored by cluster and splited by treatment (vehicle vs. trametinib treated). (C) Stacked barplot representing the proportion of each CD8 cluster cell subset (TCF1^+^, glycolytic, effector). (D) Trajectory analysis using Monocle 3, on the left UMAP showing cells colored by cluster assignment, on the right: UMAP underlining the trajectory - starting at the bottom left and ending at the top right - which defines the pseudotime coordinate of each cell. (E) Relative expression of main marker genes for each cell along pseudotime and colored by cluster. (F) Gene signatures from a set a gene up regulated in CD8+TCF1+ cells as reported in viral and tumoral models of persistent antigen, see Figure S8 for detail. (G–K) Analysis of T cell clonotype, (G) percentage of cells into each cluster with a unique clonotype, (H) distribution of CDR3 region length per cluster. (I) Stacked barplot representing for each cluster the proportion of cells belonging to the different clonotype groups ranging from rare to hyperexpended (J), Morosita’s overlap index for T cell receptor between CD8^+^ T cell subsets. (K) Plot representing the dominant CD8 clones across the three clusters.

By further investigating the CD8 compartment, we found three sub-clusters that we annotated based on the top most differentially expressed genes: (1) CD8^+^TCF1^+^ expressing high levels of *TCF7*, the gene encoding the transcription factor TCF1 known to be expressed by antigen-activated but non-differentiated CD8 T cells, (2) a glycolytic cluster, with cells expressing high levels of genes involved in glycolysis (*GAPDH*, *ENO1*), and (3) an effector cluster with highly expressed genes related to effector mechanisms within CD8 T cell (*GZMH*, *NKG7*, *PRF1*, and *GZMA*) (Figures 4A–4C). Next, we performed a trajectory analysis to explore the relationship among these subgroups and established a pathway connecting the CD8^+^TCF1^+^ cluster with the glycolytic and the latter with the effector, allowing visualization of the variation of gene marker’s expression for each subgroup along the established pseudotime (Figures 4D and 4F). Analysis of T cell clonotype confirmed this hypothesis, as the percentage of unique clonotype was higher in the CD8^+^TCF1^+^ cluster than in the glycolytic and finally in the effector clusters (Figures 4G–4I). Besides, analysis using the Morisita’s overlap index revealed high clonotype overlap between the glycolytic and the effector T cell subsets, moderate overlap between the glycolytic and the CD8^+^TCF1^+^ subsets and low overlap between CD8^+^TCF1^+^ and effector subsets (Figure 4J) as otherwise demonstrated by the pattern of dominant clone distribution (Figure 4K). These data support a clonal expansion dynamic along the CD8 lineage. Thus, inhibition of CD8 T cell differentiation toward graft-damaging effector cells during the allo-immune response may explain the preventive effect of trametinib on skin graft rejection.

To further investigate the nature of the CD8^+^TCF1^+^ cluster, we assessed the expression levels of genes known to be upregulated in stem-like or pre-exhausted CD8^+^TCF1^+^ cells, as reported in models of persistent antigen exposure (viral or tumoral). Compared to the other CD8^+^ clusters, the CD8^+^TCF1^+^ cluster exhibited significantly higher expression of these genes (Figure S8) and a correspondingly higher composite gene score (Figure 4F).^14^

**Figure 5:**
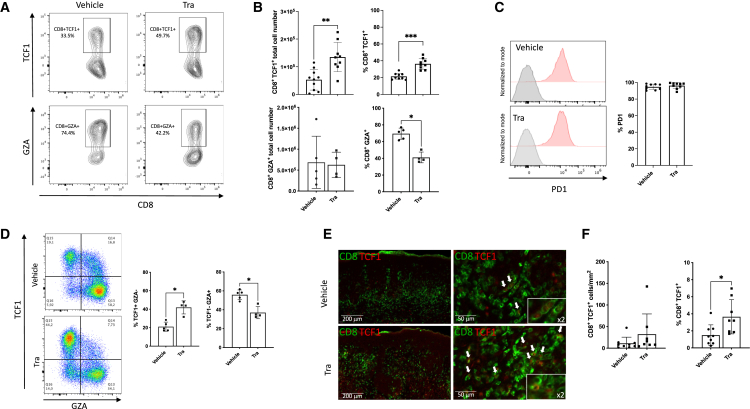
Both flow cytometry analysis of spleens and immunofluorescence of skin grafts at Day 14 post PBMC infusion show enrichment in CD8^+^TCF1^+^ in trametinib-treated animals Human skin grafts and spleens were harvested from transplanted NSG mice 14 days after injection of allogeneic hPBMCs. Mice were treated with trametinib or vehicle from days 0–14. (A) Representative contour plots of flow cytometry analysis showing the expression of TCF1 or Granzyme A in CD8 T cells splenocytes of vehicle- or trametinib-treated animals. (B) Bar plots showing total cell number and percentage of CD8^+^TCF1^+^ and CD8^+^GZA^+^ in splenocytes of vehicle- or trametinib-treated animals. Data were from four distinct experiments for CD8^+^TCF1^+^ (vehicle-treated group *n* = 10, trametinib-treated group *n* = 8) and from two distinct experiments for CD8^+^GZA^+^ (vehicle-treated group *n* = 5, trametinib-treated group *n* = 4). (C) Representative histogram of PD1 expression in CD8^+^TCF1^+^ T splenocytes of vehicle of trametinib treated animals. Bar plot showing percentage of PD1^+^ cells in CD8^+^TCF1^+^ T splenocytes. Data were from four independent experiments (vehicle-treated group *n* = 10, trametinib-treated group *n* = 8). (D) Representative flow cytometry plots showing TCF1 and granzyme A expression in splenic CD8^+^ T cells from vehicle- or trametinib-treated mice., Bar plot showing percentage of CD8^+^TCF1^+^GZA^−^ and CD8^+^TCF1^−^GZA^+^ in splenocytes of vehicle- or trametinib-treated animals. Data were from two independent experiments (vehicle-treated group *n* = 5, trametinib-treated group *n* = 4). (E) Representative images showing fluorescence patterns of CD8 (green, membranous) and TCF1 (red, intranuclear) in human skin grafts with different scales, in vehicle- or trametinib-treated animals. White arrow highlights double-positive CD8^+^TCF1^+^ cells. (F) Bar graph showing quantification of double-positive CD8^+^TCF1^+^ in vehicle or trametinib-treated animals. Pooled data of 4 experiments, vehicle treated group *n* = 9, trametinib treated group *n* = 8). (B, C, D, and F) Data are shown as means ± SD, Mann-Whitney test. ∗*p* < 0.05.

To validate this hypothesis at the protein level, we performed flow cytometry on CD8^+^ T cells isolated from spleens after 14 days of treatment. This included intracellular staining for TCF1, specifically expressed by the CD8^+^TCF1^+^ cluster, and granzyme A (GZA), characteristic of effector and glycolytic cluster cells. All CD8^+^TCF1^+^ cells expressed PD-1, distinguishing them from naive CD8^+^ T cells and supporting their classification as stem-like or pre-exhausted (Figure 5C). As expected, trametinib-treated animals exhibited a significant increase in CD8^+^TCF1^+^ cells and a decrease in CD8^+^GZA^+^ cells (Figures 5A and 5B). Figure 5D further illustrates how TCF1 and GZA staining delineate distinct CD8^+^ T cell populations, consistent with the clustering observed in the scRNA-seq data. To corroborate our findings within the skin infiltrate, we performed intracellular TCF1 staining by immunofluorescence on the human skin graft to detect CD8^+^TCF1^+^ T cells. We clearly identified cells double-positive, for CD8 on their membrane and for TCF1 in their nucleus (Figure 5E). Using a semi-automated method to quantify these CD8^+^TCF1^+^ cells, we observed that their proportion was significantly higher in the skin infiltrate of trametinib-treated animals (1.5% ± 1.2% vs. 3.6% ± 2%, *p* < 0.05) (Figure 5F).

### Trametinib promotes an IL7R-high CD4 T cell subset consistently with enhanced homeostatic reconstitution

**Figure 6:**
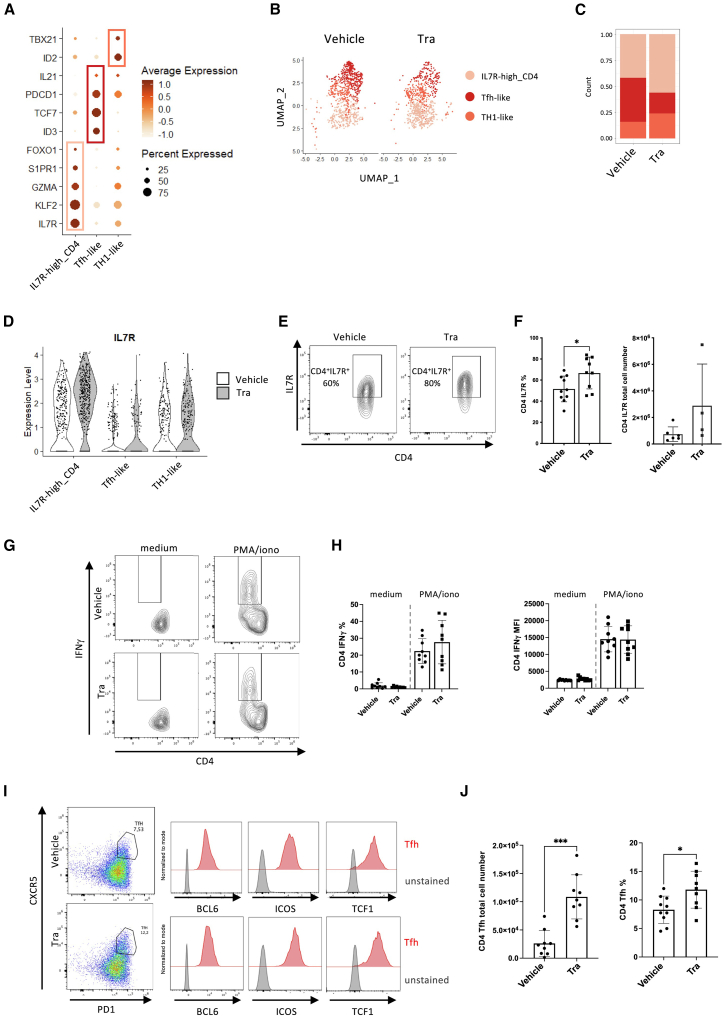
An IL7R-high subset within the CD4 compartment is promoted by trametinib treatment (A) Dot plot depicting the expression profiles (average normalized expression and percentage) of genes identified as markers based on differential expression analysis, across the three CD4 clusters. (B) UMAP projection restricted to the CD4 subset, colored by cluster and split by treatment (vehicle vs. trametinib). (C) Stacked bar plot showing the proportion of each CD4 cluster (IL7R-high, Tfh-like, TH1-like). (D) Violin plot showing the relative expression of IL7R in each CD4 cluster according to their exposure to trametinib or vehicle. (E and I) Spleens were harvested from transplanted NSG mice at day 14 after the injection of allogeneic hPBMCs. Mice were treated with trametinib or vehicle from day 0 to day 14. (E and F) For IL7R staining, results are pooled data from 2 to 4 experiments, vehicle-treated group *n* = 5 to 9, trametinib-treated group *n* = 4 to 8. (E) Representative contour plots showing expression of IL7R in splenocytes CD4 T cells in vehicle or trametinib treated animals. (F) Bar plots showing the percentage and the total cell number of CD4^+^IL7R^+^ in the splenocytes of vehicle- or trametinib-treated animals. (G and H) Splenocytes were cultured in medium alone or were stimulated with PMA-ionomycin in the presence of Brefeldine A for 4 h and stained for detection of IFNg. (G) Representative contour plots showing IFNg expression in CD4 T cells. (H) Bar plots showing the percentages of IFNg expressing CD4 T cells and the mean fluorescence intensity of IFNg expression in vehicle- or trametinib-treated animal. Results are pooled data from 4 experiments, vehicle-treated group *n* = 10, trametinib-treated group *n* = 9. (I) Representative flow cytometry plots of the gating strategy of CXCR5^+^ PD1^+^ Tfh cells in splenocytes CD4 T cells from vehicle- or trametinib-treated mice. Histogram overlays showed expression of BCL6, ICOS, and TCF1 in Tfh cells from vehicle- or trametinib-treated mice. (J) Bar plots showing total cell number and percentage of Tfh cells in splenocytes of vehicle- or trametinib-treated animals. Results are pooled data from 4 experiments, vehicle-treated group *n* = 10, trametinib-treated group *n* = 9. (F, H, and J) Data are shown as means ± SD, non-parametric Mann-Whitney test. ∗*p* < 0.05, ∗∗∗*p* < 0.001.

Concerning the CD4 T cell compartment, clustering identified three subgroups. Firstly, a population, designed as IL7R-high because it’s high expression of *IL7R* along with *FOXO1*, *KLF2*, and *S1PR1* genes (Figure 6A). *FOXO1* and *KLF2* are part of a transcriptional program that regulates the homeostasis of naive T cells by increasing IL7R, rendering cells more sensitive to homeostatic proliferation, and facilitating their trafficking through S1PR1.^15^ Trametinib-treated animals were enriched in the IL7R-high cluster and remarkably, cells exposed to trametinib within this cluster expressed higher levels of IL7R (Figures 6B–6D). Flow cytometry confirmed that the proportion of splenic CD4 T cells harvested on day 14 expressing IL7R on their membrane was significantly higher in trametinib-treated animals (Figures 6E and 6F). With the finding that trametinib-treated animals exhibit a significantly higher number of spleen CD4^+^ T cells (Figure 1C), these results collectively suggest that trametinib promotes the homeostatic reconstitution of CD4 T cells by enhancing or maintaining Il7R expression. Two other clusters were identified: a first one that appeared to be on the path of differentiating toward a follicular helper T cell phenotype (Tfh-like), since it expresses high levels of *PDCD1*, *Il21*, and the transcription factors *TCF7* and *ID3* (Figure 6A), commonly reported in CD4 T cells skewed toward the Tfh lineage.^16^^,^^17^ The second cluster, annotated as Th1-like, exhibited high levels of *TBX21* and *ID2* (Figure 6A), both transcription factors previously identified as increased in CD4 T cells belonging to the TH1 lineage.^16^^,^^18^ We confirmed at the protein level the presence of CD4^+^ T cells producing IFN-γ after stimulation, indicative of a Th1 phenotype (Figure 6G). Additionally, we observed CD4^+^CXCR5^+^PD-1^+^BCL6^+^ cells consistent with a Tfh phenotype. Notably, these cells were also TCF1 positive, supporting our annotation, as Tcf7 expression was detected in the Tfh-like cluster (Figure 6H).

At the quantitative level, although trametinib-treated animals showed an enrichment of the Th1-like cluster relative to Tfh-like, suggesting a shift in CD4^+^ T cell commitment toward the Th1 lineage, this was not confirmed at the protein level. We observed no difference in the frequency of IFN-γ-producing CD4^+^ T cells (Figure 6G), and, in contrast with scRNAseq, significantly more CD4^+^CXCR5^+^PD-1^+^BCL6^+^ Tfh cells were present in trametinib-treated animals (Figure 6H).

## Discussion

In a pre-clinical transplantation model involving human skin and allogeneic human immune cells, we found that trametinib treatment delayed rejection. While no difference in the extent of graft infiltration, skin human epidermal cells were transiently spared by the treatment. Trametinib prevented CD8 T cells from progressing to an effector phenotype, leading to a concomitant accumulation of CD8^+^TCF1^+^ stem-like cells. In addition, trametinib appeared to enhance CD4 T cell recovery by sustaining high IL7R expression.

The MEK-ERK pathway is known to be involved downstream of the TCR. Specifically, *in vitro* inhibition of MEK has been shown to selectively impair naive T cells proliferation compared with differentiated T cells.^9^ This feature might explain the efficacy of MEK inhibition in preventing GVHD in mouse models of bone marrow transplantation.^9^^,^^10^ In the context of solid organ transplantation, a significant proportion of previously primed and differentiated alloreactive T cells, especially CD8, may recognize donor MHC molecules via the direct or semi-direct pathway of allorecognition. Consistent with this, trametinib was effective only in preventing a so-called delayed rejection (attributed to indirect presentation of the alloantigen to the recipient’s naive T cells) in a rat model of lung transplantation, but did not stop early rejection.^11^ In our model of human MHC-mismatched skin transplantation, we can assume that rejection is driven by both naive and previously differentiated alloreactive T cells. Our finding of impaired CD8 T differentiation by trametinib is consistent with findings from GVHD models and may explain the partial protection we observed.

More specifically, we were able to decipher the trajectory of CD8 T cells following their activation. They went from an activated TCF1^+^ subset to an effector state through an intermediate state exhibiting an enhanced glycolytic that might have driven the CD8 differentiation.^19^^,^^20^ While TCF1 is typically expressed in naive CD8 T cells, its sustained expression following activation is unusual, except in some cells that retain a high potential of proliferation. Notably, these CD8^+^TCF1^+^ cell have been described in both mice and humans in the contexts of persistent infection and cancer. They have been termed stem-like^21^^,^^22^ or Tpex for precursors of exhausted T cells,^23^ highlighting that, contrary to the exhausted T cells, they keep a strong potential of expansion and are able to convert themselves to effector TCF1^-^ T cells. Recently, assuming that the allo-immune response and persistent infection are both driven by the persistence of high antigen levels, Lee et al.^24^ proposed that TCF1^+^CD8^+^ could be similarly generated in both situations. By directly comparing with chronic LCMV infection, they confirmed the presence of CD8^+^TCF1^+^ cells in an MHC-mismatched mouse model of acute GVHD, as well as in a xenogeneic GVHD model using NSG mice reconstituted with hPBMC. Our study is the first to report this CD8^+^TCF1^+^ subset in the context of solid organ transplantation. Notably, their transcriptional signature, as assessed by scRNA-seq, closely resembled profiles previously reported in chronic viral infection and tumor models.^14^

In our model, trametinib-treated animals show an enrichment of the TCF1^+^ subset, suggesting that MEK inhibition may have impaired their differentiation into TCF1^-^ effector cells. This observation is consistent with findings in tumor models, where trametinib was shown to impair the priming of tumor-specific naive CD8 T cells while promoting the accumulation of Tpex CD8^+^TCF1^+^ by impairing their evolution into exhausted CD8^+^TCF1^-^ cells.^25^^,^^26^

In the context of clinical renal transplantation, converging evidence points to the detrimental role of CD8 terminally differentiated (TEMRA) on the transplanted kidney. Indeed, their importance at 1-year post-transplantation as well as in the graft infiltrate has been found to be independently associated with graft loss.^27^^,^^28^ Importantly, CD8^+^TCF1^+^ might be the source of alloreactive TEMRA after their differentiation to CD8^+^TCF1^-^, similar to what has been established in anti-tumor or anti-viral immunity.

Although further investigation will be required to determine the precise role of CD8^+^TCF1^+^ in the setting of organ transplantation, our finding that they are represented in a humanized model of skin allograft and that trametinib affects their conversion to differentiated CD8, a finding confirmed in the graft infiltrate, is particularly relevant.

Regarding the CD4 T cell compartment, we found that trametinib-treated animals not only exhibited an increase in a subset of cells expressing IL7R, but that within this IL7R-high CD4 subset, IL7R was one of the few genes differentially over-expressed. IL-7 plays a pivotal role in T cell homeostasis. Under normal physiological conditions, IL-7 supports the renewal of naive and memory T cells by promoting the capture of available IL-7 in the environment, a process accompanied by downregulation of its receptor, CD127, ensuring self-limiting proliferation.^29^ In our model, we can assume that the immune reconstitution after hPBMC infusion is driven by the allo-immune reactivity of a fraction of lymphocytes, but also by lymphopenia-induced proliferation, a process analogous to the homeostatic proliferation occurring after lymphocyte adoptive transfer into a lympho-depleted host.^30^ A limitation of our model is that we were not able to discriminate between allo-reactive and non-alloreactive lymphocytes. However, since the transcriptional signature of the IL7R-high CD4 T cell was indicative of cells undergoing homeostatic proliferation,^15^ we may hypothesize that trametinib promoted this phenomenon. The CD4/CD8 ratio increase in the spleen of trametinib treated animals suggests that this effect is predominant in CD4 over CD8 T cells. This may be explained by the fact that homeostatic proliferation of CD8 T cell requires IL-15 in addition to IL-7, whereas IL-7 alone is sufficient for CD4 T cell.^31^ While mouse IL-7 cross-reacts with human IL7R,^32^ mouse IL-15 does not.^33^ While we observed a defect in the IL7R downregulation in the CD4 from treated animal, its relationship with the inhibition of the MEK-ERK pathway remains to be determined. In particular, neither TCR nor IL2R downstream signaling, both of which lead to IL7R downregulation, appear to be dependent on this pathway.^34^^,^^35^ Notably, a similar effect on promoting T cell reconstitution, had previously been observed in a model of xenoreactivity, without any insight into its mechanisms.^36^

Our findings, when transposed into the context of clinical transplantation, raise the question whether this property would persist and prove beneficial in transplant recipients with persistent anti-lymphocyte polyclonal Ab-induced lymphopenia and simultaneously receiving other immunosuppressive therapies. Indeed, prolonged lymphopenia has been associated with death and graft failure in the setting of kidney transplantation.^37^^,^^38^

Regarding the effect of trametinib on CD4^+^ differentiation toward a Th1 or Tfh fate, we observe a discrepancy between the scRNAseq data and the phenotype in flow cytometry. While scRNAseq data suggest that trametinib favor Th1-like over Tfh-like, we observe significantly more CD4^+^CXCR5^+^PD-1^+^BCL6^+^ Tfh by flow cytometry. Several factors could explain this discrepancy. First, the difference in the scRNA-seq experiment is modest, and the sample size was limited (*n* = 2 for each group). Second, the Tfh-like cluster that we identified using an unbiased approach may not fully capture the Tfh phenotype as assessed by flow cytometry. Finally from a theoretical standpoint, there are arguments suggesting that trametinib may inhibit shift toward a Tfh-like identity, as IL-7 signaling has been shown to repress the Tfh gene program, in particular through the downregulation of KLF2 and SR1P1^39^^,^^40^ which we are highly expressed in the CD4-IL7Rhigh cluster. Alternatively, a study by Wan et al.^41^ showed that *in vitro*, MEK inhibition promotes a Tfh-like transcriptomic profile by directly upregulating transcription factors such as Bcl6 and TCF1, as ERK acts as a repressor of Tfh differentiation. Therefore, we currently lack sufficient evidence to conclusively determine how trametinib influences CD4^+^ T cell differentiation in our model.

In conclusion, our findings demonstrate that pharmacological MEK inhibition delays the rejection of human skin grafts in a preclinical humanized mouse model. This protective effect appears to be achieved by suppressing the differentiation of CD8 T cells and promoting the accumulation of CD8^+^TCF1^+^ cells, both in the spleen of the animals and within the human skin allografts. Furthermore, treatment with MEK inhibitors may promotes the recovery of CD4 T cells, a potentially valuable outcome in the management of immunosuppressive treatment-induced lymphopenia. These observations point to the potential utility of MEK inhibitors in modulating immune responses and preserving graft function, though further investigation is needed to validate these effects in clinical settings.

### Limitations of the study

While the NSG humanized mouse model offers the advantage of using human immune cells, it also has limitations. The model is not antigen-specific, which limits the distinction between how trametinib affect homeostatic reconstitution and alloimmune responses. Additionally, the inherent kinetics of immune reconstitution, combined with variability between donors, adds another layer of complexity. This limits precise manipulation within the system, which would be necessary to definitively demonstrate the impact of trametinib on immune reconstitution and alloimmune responses.

## Resource availability

### Lead contact

Further information and requests for resources and reagents should be directed to and will be fulfilled by the lead contact, Simon Ville (simon.ville@chu-nantes.fr).

### Materials availability

This study did not generate any unique reagent.

### Data and code availability

• ScRNA-seq data generated in this study have been deposited in the NCBI Gene Expression Omnibus (GEO) and are accessible through accession number GSE266482.
• This paper does not report original code.
• Any additional information required to reanalyze the data reported in this paper is available from the lead contact upon request.

## Acknowledgments

This work was supported by the INSET from Region Pays de la Loire. We thank the humanized Rodents Platform from the Labex IGO in Nantes. The humanized rodent platform was supported by the Labex IGO project (*n*° ANR-11-LABX-0016-01) funded by the Programme d’Investissements d’Avenir, which is a French Government program managed by the 10.13039/501100001665French National Research Agency (ANR). We thank the Genomics and Bioinformatics core facility of Nantes (GenoBiRD, Biogenouest, IFB) for its technical support. We also thank the IBISA MicroPICell facility (Biogenouest), member of the national infrastructure France-Bioimaging supported by the 10.13039/501100001665French National Research Agency (ANR-10-INBS-04). The authors thank Sophie Limou **(**Center for Research in Transplantation and Translational Immunology**,** UMR 1064, *University of Nantes) for her critical reading of the manuscript.* Schematic diagrams of experimental design were created with Biorender.com.

## Author contributions

Conceptualization, C.C. and S.V.; methodology, C.C., V.N.-D. and M.F.; investigation, C.C., F.H., G.B., and S.V.; single cell experiment: C.F., L.B., M.B., J.P., and S.V.; resources, T.L., L.D., O.R., and J.V.; supervision, C.C. and S.V.; funding acquisition, C.C., G.B., and S.V.; writing – original draft: C.C, and S.V.; writing – review and editing, all authors.

## Declaration of interests

The authors declare no conflict of interest.

## STAR★Methods

### Key resources table

**Table:** 

REAGENT or RESOURCE	SOURCE	IDENTIFIER
**Antibodies**		

Anti-mouse CD45 PerCpCy5-5	BD Pharmingen	Cat# 563792; RRID: AB_2869519
Anti-human CD45 BUV 395	BD Biosciences	Cat# 563792; RRID: AB_2869519
Anti-human CD3 BV711	BD Biosciences	Cat# 563725; RRID: AB_2744392
Anti-human CD4 BV500	BD Biosciences	Cat# 560768; RRID: AB_1937323
Anti-human CD4 FITC	BD Biosciences	Cat# 555346; RRID: AB_395751
Anti-human CD4	Biolegend	Cat# 317402; RRID: AB_571963
Anti-human CD8 BV605	BD Biosciences	Cat# 569169; RRID: AB_3684836
Anti-human CD8	Abcam	Cat# ab4055; RRID: AB_304247
Anti-human CD19 V450	BD Biosciences	Cat# 560353; RRID: AB_1645564
Anti-human CD56 BV650	BD Biosciences	Cat# 742660; RRID: AB_2740951
Anti-human CD14 V500	BD Biosciences	Cat# 561391; RRID: AB_10611856
Anti-human CD45RA BV786	BD Biosciences	Cat# 563870; RRID: AB_2738459
Anti-human CD45RA BB700	BD Biosciences	Cat# 742249; RRID: AB_2871441
Anti-human CD27 V450	BD Biosciences	Cat# 560448; RRID: AB_1645567
Anti-human CD127 BUV 737	BD Biosciences	Cat# 612794; RRID: AB_2870121
Anti-human CD25 FITC	BD Biosciences	Cat# 555431; RRID: AB_395825
Anti-human PD1 PE Cy7	Biolegend	Cat# 329918; RRID: AB_2159324
Anti-human PD1 BV785	Biolegend	Cat# 329930; RRID: AB_2563443
Anti-human CD197 BV421	Biolegend	Cat# 353208; RRID: AB_11203894
Anti-human CXCR5 BV750	BD Biosciences	Cat# 747111; RRID: AB_2871862
Anti-human ICOS APC	Biolegend	Cat# 313510; RRID: AB_416334
Anti-human BCL6 PE-Cy7	Biolegend	Cat# 358512; RRID: AB_2566196
Anti-human IFNγ PE-CF594	BD Biosciences	Cat# 562392; RRID: AB_11153859
Anti-human TCF1 AF647	Biolegend	Cat# 655204; RRID: AB_2566620
Anti-human TCF1	Biolegend	Cat# 655202; RRID: AB_2562103
Anti-human Granzyme A PE Cy7	Biolegend	Cat# 507222; RRID: AB_2721668
Anti-human FoxP3	Thermofisher	Cat# 14-4777-82; RRID: AB_467556
Anti-Ki67	Abcam	Cat# ab15580; RRID: AB_443209
Goat anti-mouse IgG1 AF568	Invitrogen	Cat# A-21124; RRID: AB_2535766
Goat anti-mouse IgG2b AF488	Invitrogen	Cat# A-21141; RRID: AB_2535778
Donkey anti-rabbit IgG (H + L) AF488	Jackson Immunoresearch	Cat# 711-545-152; RRID: AB_2313584
Goat anti-rabbit IgG HRP	Jackson Immunoresearch	Cat# 111-035-003; RRID: AB_2313567

**Biological samples**		

Blood samples	Etablissement Français du sang	N/A

**Chemicals, peptides, and recombinant proteins**		

Trametinib	Clinisciences	Cat# HY-10999
Phorbol 12-myristate 13-acétate	Sigma	Cat# P8139
ionomycin	Sigma	Cat# I9657
Brefeldine A	Sigma	Cat# B6542
Live/dead Blue	Thermofisher	Cat# L34962

**Experimental models: Organisms/strains**		

NOD.Cg-Prkdc^scid^ Il2rγ^tm1Wjl^ NSG	Labex IGO	In-house colony

**Software and algorithms**		

Flowjo V10	Tree Star Inc	RRID: SCR_008520 https://www.flowjo.com/
Prism V10.4.1	GraphPad Software Inc.	RRID: SCR_002798 https://www.graphpad.com/
QuPath v0.4.3	Bankhead et al.^42^	RRID: SCR_018257 https://qupath.github.io

**Deposited data**		

ScRNA-seq data	This publication	GEO: GSE266482

### Experimental model and study participant details

#### Mice

The NOD.Cg-Prkdc^scid^ Il2rγ^tm1Wjl^ immunodeficient mice (NSG, the Jackson Laboratory, Bar Harbor, USA) were bred by the LabEx IGO Humanized rodent Platform in an accredited animal facility (accreditation C44-278). All animals were housed under specific pathogen–free conditions, at controlled temperature (22 ± 2 °C) and 12 h light/dark cycle. Mice, female or male, were between 7 and 10 weeks-old at inclusion. All experiments were performed according to authorization APAFIS #22146 and #34276 from French Ministry of Research.

#### Cells

hPBMC from buffy coats were collected at *Etablissement Français du Sang* (Nantes, France) from healthy donors (HD). Written informed consent was provided according to institutional guidelines. hPBMCs were isolated by Ficoll-Paque density-gradient centrifugation (Eurobio, France).

### Method details

#### Human skin transplantation

Caucasian human skin samples were obtained from HD with informed consent, in accordance with institutional guidelines. The transplantation procedure was performed as previously described.^43^ Briefly, a 1 × 1 cm piece of shaved mouse skin was removed from the dorsal thorax over the costal margin and replaced by a 1 × 1 cm piece of human skin sutured to the mouse recipient skin using nonabsorbable 8-0 polypropylene (Ethicon). Grafts were covered with mesh and a pressure dressing and secured with circumferential tape. Bandages remained for 7–9 days before being removed.

#### Humanized mouse model

Seven weeks after the skin transplantation, 5 to 10.10^6^ allogeneic hPBMCs were injected intravenously (i.v). Human chimerism was monitored weekly in blood by flow cytometry and calculated as follows: (% of hCD45)/(% of hCD45 + % of mCD45) x 100. Body weight, fur appearance and posture of the animals were monitored daily. For each combination human skin donor/allogeneic human PBMCs donor, animals were allocated to two groups: one was treated with trametinib, while the other received a sham treatment as a control.

#### Treatment

Trametinib (MedChemExpress) was reconstituted in DMSO (Sigma), stored at −80°C and diluted in methylcellulose 0.5% (Sigma) for treatment according to published methods.^11^ In all experiments, trametinib (0.9 mg/kg) or sham treatment (*i*.*e*., methylcellulose 0.5%) were orally administered every day from adoptive cell transfer.

#### Human skin graft monitoring

Following the adoptive transfer of hPBMCs, each skin graft was monitored daily in a blindly manner and visually scored on a scale from 0 to 4 (Figure S5A). Grafts scoring 3 or higher were considered rejected, as per established criteria. Additionally, 15 mice were euthanized 14 days post-transfer to further assess signs of skin rejection and inflammation at a single time point.

#### Flow cytometry

For *in vivo* experiments, blood was collected weekly and at endpoints. Spleen from euthanized mice were mechanically dissociated and filtered through 100 μm nylon filters (Falcon Biosciences). Ammonium chloride potassium buffer was used for red cell lysis. For cell surface staining, cells were labeled with antibodies 30 min at 4°C. eBioscience TM Foxp3/Transcription Factor Staining Buffer Set (ThermoFisher) was used for intracellular staining. Flow cytometry was performed using FACS Celesta (BD Biosciences) or Aurora (Cytek) and analyzed using the FlowJo software (Tree Star).

#### Intracellular IFNγ measurement

To assess IFN-γ expression in splenic CD4^+^ T cells, 1×10^6^ splenocytes were cultured in complete RPMI medium supplemented with 10% fetal calf serum (FCS), 100 U/mL penicillin, and 100 U/mL streptomycin. Cells were either left unstimulated or stimulated for 4 h with PMA (500 ng/mL) and ionomycin (5 μg/mL) in the presence of brefeldin A (5 μg/mL). Following stimulation, cells were stained for surface markers (hCD45, hCD3, hCD4, and hCD8), then fixed, permeabilized, and stained intracellularly for IFN-γ. Flow cytometric analysis was performed on a Cytek Aurora (Cytek Biosciences), and data were analyzed using FlowJo software (Tree Star).

#### Histopathological analysis

Lesions corresponding to skin allograft rejection were graded on hematoxylin and eosin (H&E)-stained skin slides by a board-certified pathologist (T. Larcher) in a blinded fashion using a modified previously published Banff classification.^44^^,^^45^ (Figure S5B).

#### Immunohistochemistry

Human skin grafts and surrounding mouse skin were harvested 14 days after adoptive cell transfer. Immunofluorescence and colorimetric staining were performed on 10 μm acetone-fixed frozen skin sections. After blocking non-specific binding with normal goat serum (NGS), and endogenous peroxidases with 3% H2O2 for colorimetric staining only, a mixture of primary antibodies was incubated overnight at 4°C in PBS with BSA 4% and saponin 0.1% to permeabilize the section. After three PBS washes, a mixture of secondary antibodies was applied to the section to characterize the cellular infiltrate. Colorimetric reaction was revealed with Novared kit (Vector Laboratories). Images were captured with Nanozoomer HAMAMATSU and analyzed using QuPath software.^42^ The Ki67 index represents the percentage of Ki67-positive cells among the total cells (DAPI positive).

#### Single-cell RNA-sequencing

Four humanized skin-transplanted mice were used, two treated with trametinib and two with sham treatment. Fourteen days later, spleens were harvested and flushed prior being enriched for hCD45 positive and DAPI negative cells by FACS sorting (FACS ARIA, BD Biosiences). Before mixing all cells together, we performed a cell hashing strategy using CITE-seq technology^46^ with appropriate antibodies tagged with different oligonucleotides assigned to each sample (# Hashtags anti-humanTotalseq-C, Biolegend). Cells were resuspended at the recommended dilution for 20000 cells, loading onto a Next GEM Chip K (PN-1000286) and run-on Chromium Single Cell Controller using the Chromium Single Cell 5′ V2 Next GEM single cell kit (PN-1000263) with 5′ feature barcode technology (PN-1000541) according to the manufacturer’s instructions (CG000330, 10× Genomics). Gex, Cell Surface and VDJ-TCR libraries were prepared and sequenced on an S1 flow cell on a Nova-Seq 6000 (Illumina) at the GenoBiRD platform (IRS-UN, CHU Nantes). Raw reads were produced with bcl2fastq2 (v2.20), analyzed using FastQC for quality controls and processed using the CellRanger pipeline (v7.0.0) with default parameters. Generated FASTQ files were aligned to the human reference genome GRCh38.

#### scRNA-seq data processing and analysis

The data analysis was performed using the Seurat v4 R package. Briefly, we demultiplexed the samples to identify cells from the treated animals from those from the sham animals, while at the same time eliminating potential doublets. We performed the classical Seurat pipeline for quality controls (nFeature_RNA >200 and nCount-RNA <20,000 and percent.mt < 5%), normalization, and identification of most variable genes (using vst, *n* = 2000). Seurat cell cycle scoring and regression strategy were used to reduce the effect of cell cycle phases. We resumed the usual pipeline, applying a PCA and clustering the cells. We proceeded to annotate each cluster by first looking at known marker genes to distinguish between the CD4, CD4 Treg, CD8 and B cells. Then, we investigated marker genes using differential gene expression within the CD4 or CD8 compartments. For the CD8 T cell specifically, we used Monocle 3 according to the pipeline available online to build cell trajectories and set pseudotime for each cell, allowing us to visualize gene expression along it. Finally, we analyzed CD4 and CD8 TCR repertories using the *scRepertoire* package.

### Quantification and statistical analysis

Survival data were analyzed using the log rank test. The *p* value of Banff scores was determined using the Fisher’s exact test. Other statistical analyses were conducted using GraphPad Prism version 10 (GraphPad Software Inc, San Diego, CA). Values shown in bar graphs represent mean ± standard error of the mean. Differences between unpaired groups were analyzed with the non-parametric Mann-Whitney test and were considered significant when *p* < 0.05.
