# Supplementary material for: MEK inhibition prevents human skin graft rejection by promoting CD8+TCF1+ over CD8 effector T cells
Source: iScience. 2025 Aug 6;28(9):113310. doi: 10.1016/j.isci.2025.113310 (PMC12397921; doi:10.1016/j.isci.2025.113310)
Supplement: Document S1. Figures S1–S8 [file mmc1.pdf]

iScience, Volume *28*

Supplemental information

MEK inhibition prevents human skin graft rejection by promoting CD8$^+$TCF1$^+$ over CD8 effector T cells

Christine Chauveau, Veronique Nerriere-Daguin, Maeva Fourny, Cynthia Fourgeux, Thibaut Larcher, Laurence Delbos, Martin Braud, Lucas Brusselle, Olivia Rousseau, Jeremie Poschmann, Julien Verdier, Fabienne Haspot, Gilles Blancho, and Simon Ville

Supplementary Data

Table of contents

Supplementary Figure 1. Trametinib treatment inhibits MEK pathway *in vivo*

Supplementary Figure 2. All mice maintained a stable body weight

Supplementary Figure 3. Gating strategy for splenocytes analysis.

Supplementary Figure 4. Percentages and total numbers of the cell populations present in the spleen of vehicle and trametinib-treated animals.

Supplementary figure 5. Scoring criteria of skin graft rejection

Supplementary figure 6. Quality control metrics of the entire dataset prior to any cell removal

Supplementary figure 7. Flow cytometry analysis of splenocytes harvested from vehicle and trametinib-treated animals used for single-cell RNAseq experiment

Supplementary figure 8. Expression profiles of genes identified as markers base on differential expression analysis, across the CD8 clusters

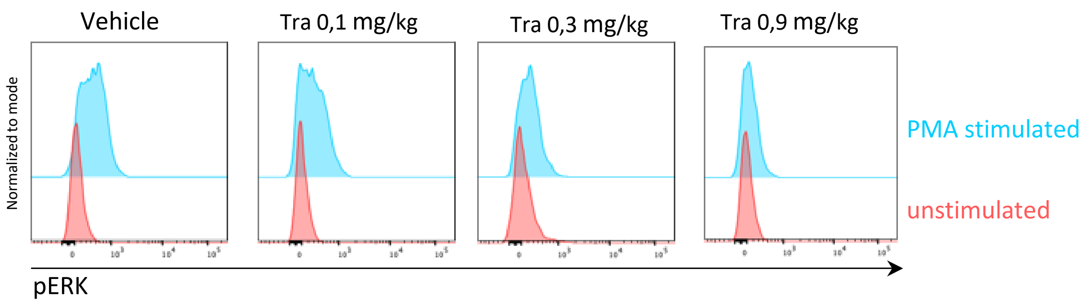

Supplementary Figure 1. Trametinib treatment inhibits MEK pathway *in vivo*
Humanized mice were orally treated daily with vehicle (n=3), trametinib (Tra) at 0.1 mg/kg (n=2), Tra at 0.3 mg/kg (n=1), or Tra at 0.9 mg/kg (n=3) for 57 days. PBMCs were harvested, stimulated for 15 minutes with 500nM PMA, and then assessed for ERK phosphorylation by flow cytometry. The figure shows representative results for one animal per condition.

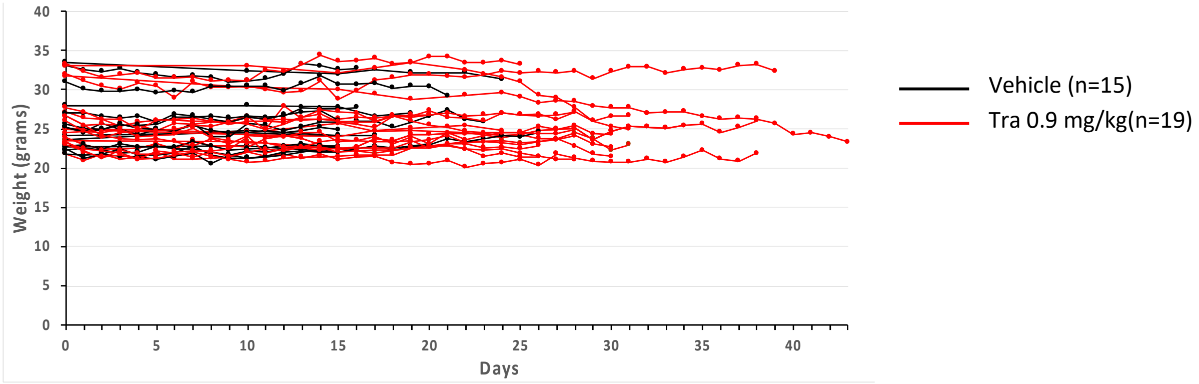

Supplementary Figure 2. All mice maintained a stable body weight
Human skin-grafted mice, subsequently humanized, were orally treated daily with either vehicle (n=15) or tra at 0.9 mg/kg (n=19) until the skin human graft was scored at 3. Body weight of the animals was evaluated daily in order to detect potential xenogeneic GVHD

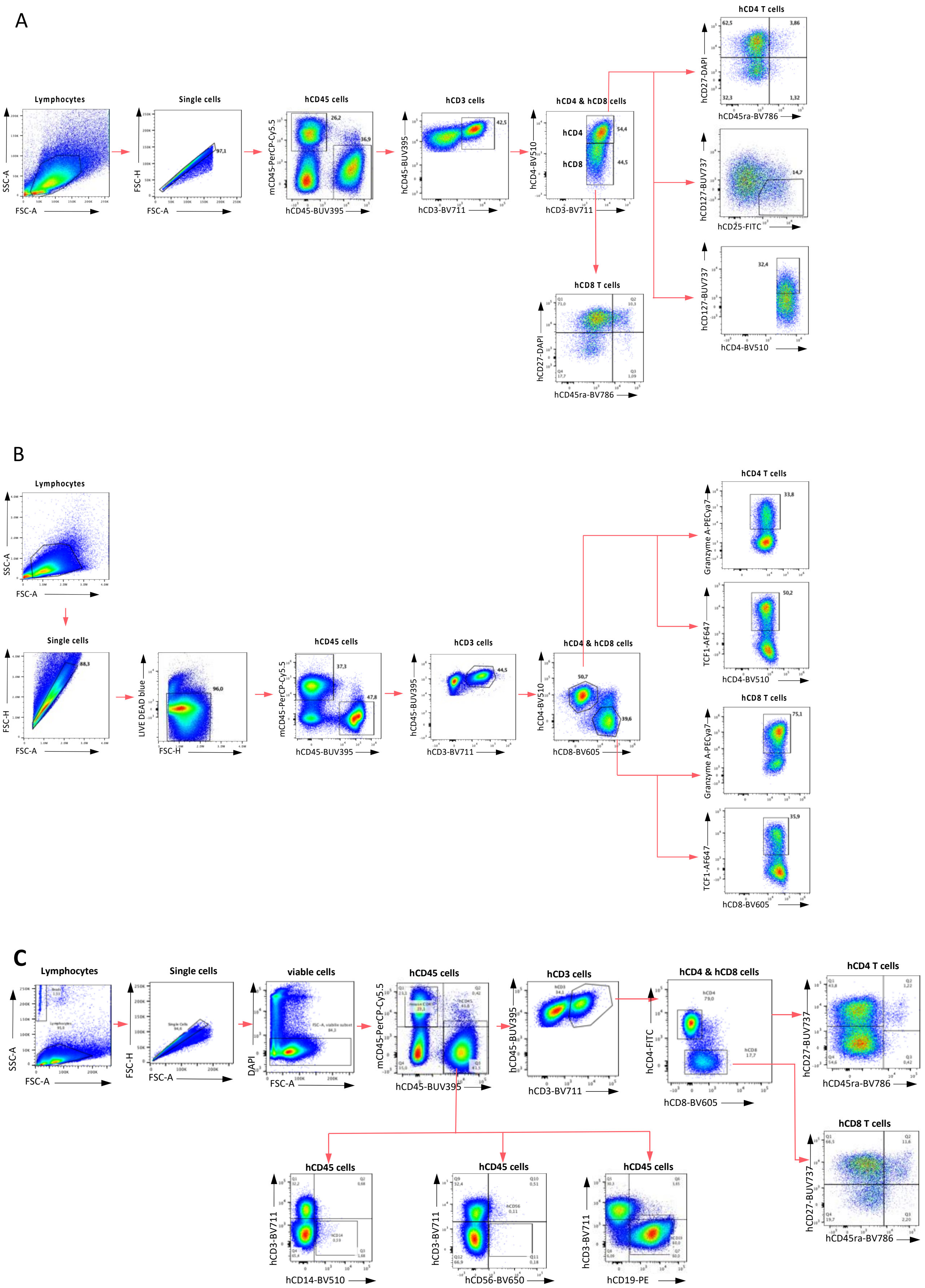

A
Lymphocytes
SSC-A
FSC-A
Single cells
FSC-H
FSC-A
hCD45 cells
mCD45-PerCP-Cy5.5
hCD45-BUV395
hCD3 cells
hCD45-BUV395
hCD3-BV711
hCD4 & hCD8 cells
hCD4
hCD8
hCD4-BV510
hCD3-BV711
hCD4 T cells
hCD27-DAPI
hCD45ra-BV786
hCD127-BUV737
hCD25-FITC
hCD127-BUV737
hCD4-BV510
hCD8 T cells
hCD27-DAPI
hCD45ra-BV786
B
Lymphocytes
SSC-A
FSC-A
Single cells
FSC-H
FSC-A
LIVE DEAD blue
FSC-H
hCD45 cells
mCD45-PerCP-Cy5.5
hCD45-BUV395
hCD3 cells
hCD45-BUV395
hCD3-BV711
hCD4 & hCD8 cells
hCD4-BV510
hCD8-BV605
hCD4 T cells
Granzyme A-PECya7
TCF1-AF647
hCD4-BV510
hCD8 T cells
Granzyme A-PECya7
TCF1-AF647
hCD8-BV605
C
Lymphocytes
SSC-A
FSC-A
Single cells
FSC-H
FSC-A
viable cells
DAPI
FSC-A
hCD45 cells
mCD45-PerCP-Cy5.5
hCD45-BUV395
hCD3 cells
hCD45-BUV395
hCD3-BV711
hCD4 & hCD8 cells
hCD4-FITC
hCD8-BV605
hCD4 T cells
hCD27-BUV737
hCD45ra-BV786
hCD8 T cells
hCD27-BUV737
hCD45ra-BV786
hCD45 cells
hCD3-BV711
hCD14-BV510
hCD45 cells
hCD3-BV711
hCD56-BV650
hCD45 cells
hCD3-BV711
hCD19-PE

Supplementary Figure 3. Gating strategy for splenocytes analysis.

(A) Splenocytes were incubated with a mixture of antibodies conjugated to fluorescent markers, including anti-mCD45, anti-hCD45, anti-hCD3, anti-hCD4, anti-hCD45RA, anti-hCD27, anti-hCD25, and anti-hCD127. Data acquisition was performed using a BD Celesta flow cytometer, and subsequent analysis was conducted with FlowJo software. Shown are representative FACS plots of a splenocyte sample and indicate strategy to analyze hCD4 and hCD8 T cell population. The relative expression of CD27 and CD45RA within the CD4 and CD8 populations identify CD45RA+CD27+ naïve cells, CD45RA-CD27+ central memory (CM) cells, CD45RA+CD27- terminal effector memory RA+ (TEMRA) cells, and CD45RA- CD27- effector memory (EM) cells. Within the CD4 population, CD127 and CD25 expression reveal regulatory T. Numbers indicate percentage of the parent population. **(B)** Splenocytes were incubated with antibodies conjugated to fluorescent markers, including anti-mCD45, anti-hCD45, anti-hCD3, anti-hCD4, anti-hCD8, anti-TCF1 and anti-granzyme A. Data acquisition was performed using a Cytek Aurora flow cytometer, and subsequent analysis was conducted with FlowJo software. **(C)** Splenocytes were incubated with a mixture of antibodies conjugated to fluorescent markers, including anti-mCD45, anti-hCD45, anti-hCD3, anti-hCD4, anti-hCD8, anti-hCD45RA, anti-hCD27, anti-hCD14, anti-hCD56 and anti-hCD19. Data acquisition was performed using a BD Celesta flow cytometer, and subsequent analysis was conducted with FlowJo software. Shown are representative FACS plots of a splenocyte sample and indicate strategy to analyze hCD4 and hCD8 T cell population. The relative expression of CD27 and CD45RA within the CD4 and CD8 populations identify CD45RA+CD27+ naïve cells, CD45RA-CD27+ central memory (CM) cells, CD45RA+CD27- terminal effector memory RA+ (TEMRA) cells, and CD45RA- CD27- effector memory (EM) cells.

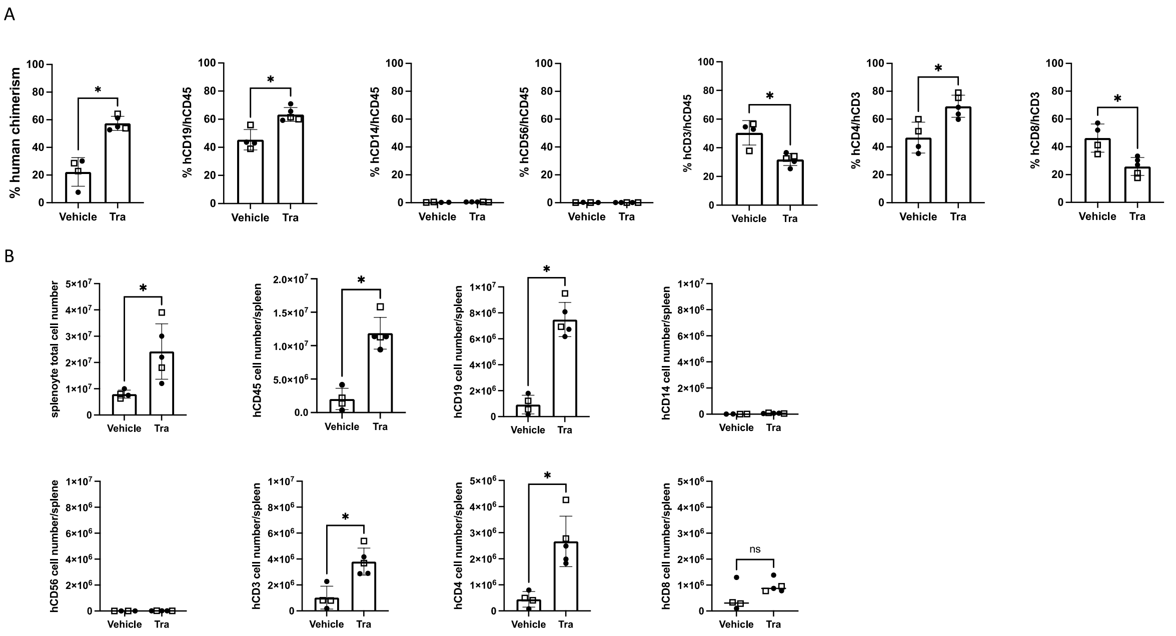

Supplementary Figure 4. Percentages and total numbers of the cell populations present in the spleen of vehicle and trametinib-treated animals.

Human cell reconstitution in the spleen 14 days after humanization was evaluated in an experiment including nine NSG mice grafted with human skin and reconstituted with human PBMCs from two different donors (□, donor 1, ●, donor 2) (vehicle-treated, n = 4; trametinib-treated, n = 5). **(A)** Bar plots showing the chimerism and the percentage of hCD19 cells, hCD14 cells, hCD56 cells, hCD3 cells, hCD4 cells and hCD8 cells in spleen of vehicle and trametinib treated mice. **(B)** Bar plots showing the total number of splenocytes and the total number of hCD45 cells, hCD19 cells, hCD14 cells, hCD56 cells, hCD3 cells, hCD4 cells and hCD8 cells in spleen of vehicle and trametinib treated mice. Data are shown as means ± SD, Mann-Whitney test. ns: non-significant, *$p < 0.05$.

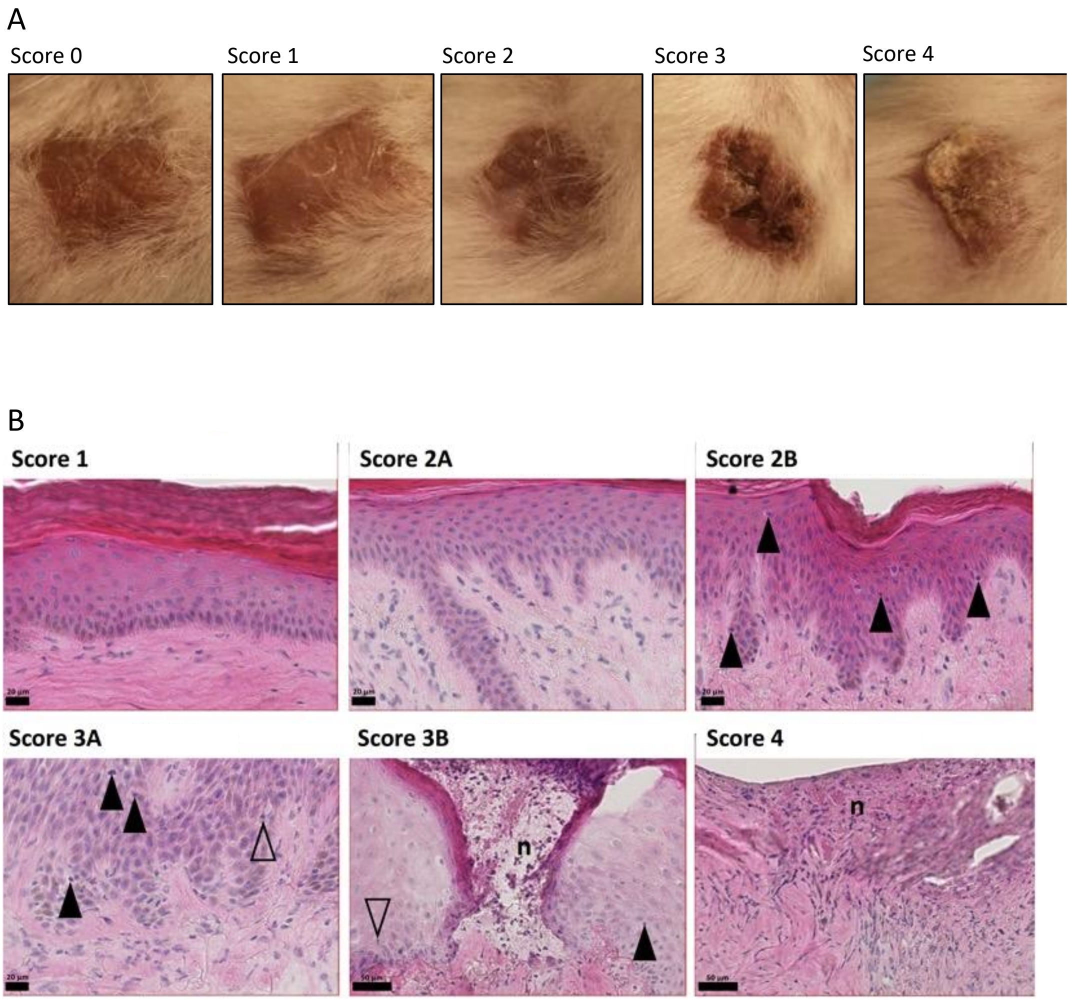

Supplementary Figure 5. Scoring criteria of skin graft rejection

(A) Representative images of skin graft rejection. Graft examinations were scored from 0 to 4 according to the following criteria: score 0, intact, soft graft with no color change; score 1, soft with redness in small areas; score 2, redness with induration in small areas; score 3, large areas of induration and redness and/or scab formation; and score 4, completely shrunken and scabbed graft, very little of original graft viable. **(B)** histopathological analysis of human skin graft rejection observed with high magnification. Lesions corresponding to skin allograft rejection were graded on H&E staining skin slides using a previously published modified Banff classification. Grade 0 consists of normal dermal and epidermal skin without evidence of inflammation. Grade 1 and grade 2A are characterized by no epidermal involvement. Grade 2B is characterized by some epidermal infiltrating inflammatory cells (▼). Grade 3A is characterized by infiltrating inflammatory cells with single cell keratinocyte necrosis (▽). Grade 3B and 4 are respectively characterized by focal and diffuse full-thickness epidermal necrosis (n).

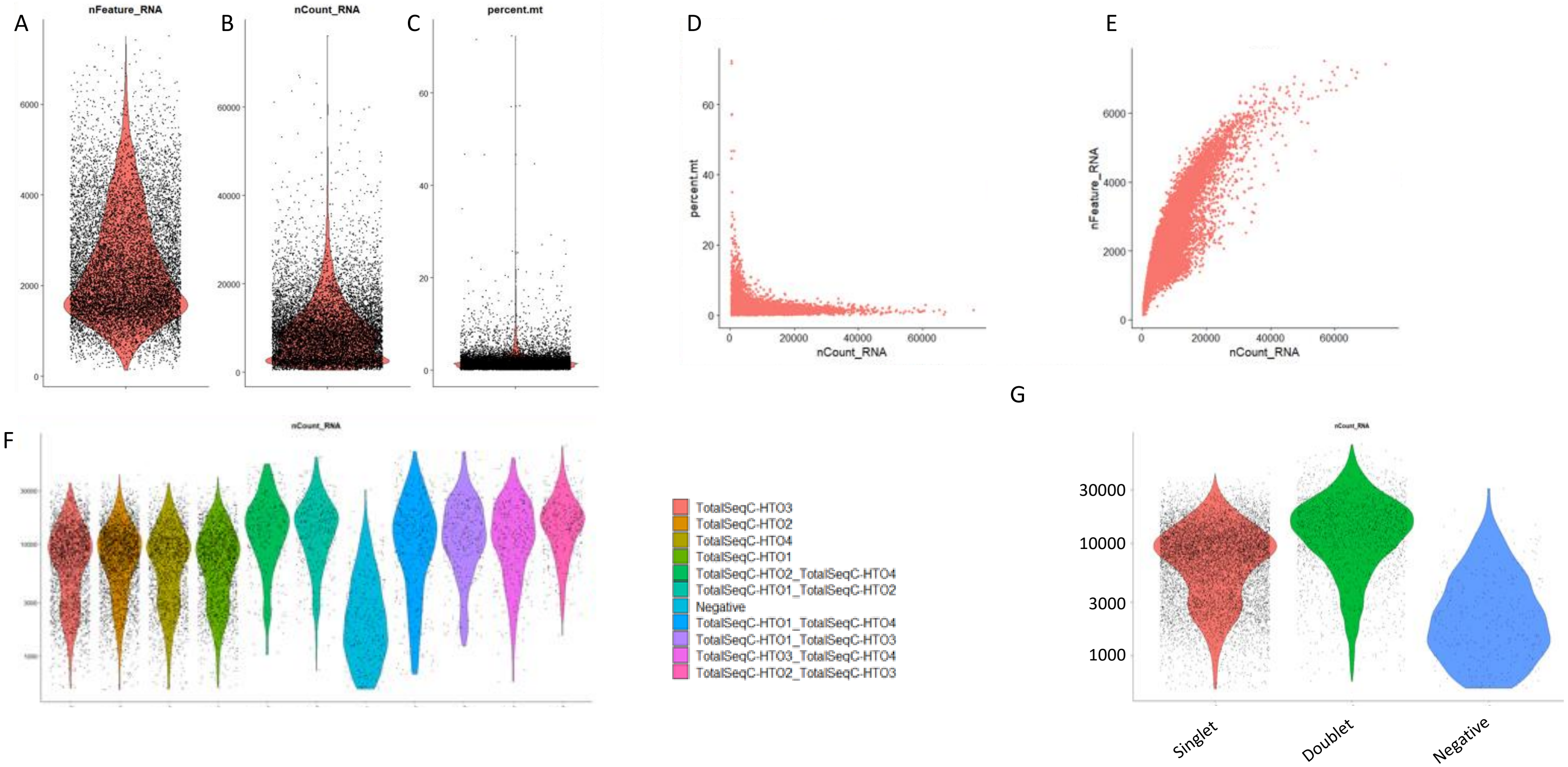

Supplementary Figure 6. Quality control metrics of the entire dataset prior to any cell removal

A-C. Violin plot showing the number of different genes captured per cell (**A**), the number of genes counted per cell (**B**) and the percentage of mitochondrial DNA per cell (**C**). **D-E**. Scatterplot showing the correlation between the number of counts per cell and the percentage of mitochondrial DNA (**D**) and between the number of different genes and the number of counts per cell (**E**). **F-G**. Violin plot showing the number of counts according to the result of demutiplexing. (**F**) The four plots on the left are singlets from the four animals (2 vehicle and 2 trametinib-treated mice) and show a remarkable homogeneity between the sample. The others are the negative droplet (probably without cell and therefore with a low number) and the droplet with two different labelled antibodies, probably a doublet, consistent with their high number. (**G**) Violin plot of the singlet, doublet and negative droplet across all samples. Negative and doublet were removed from further analysis.

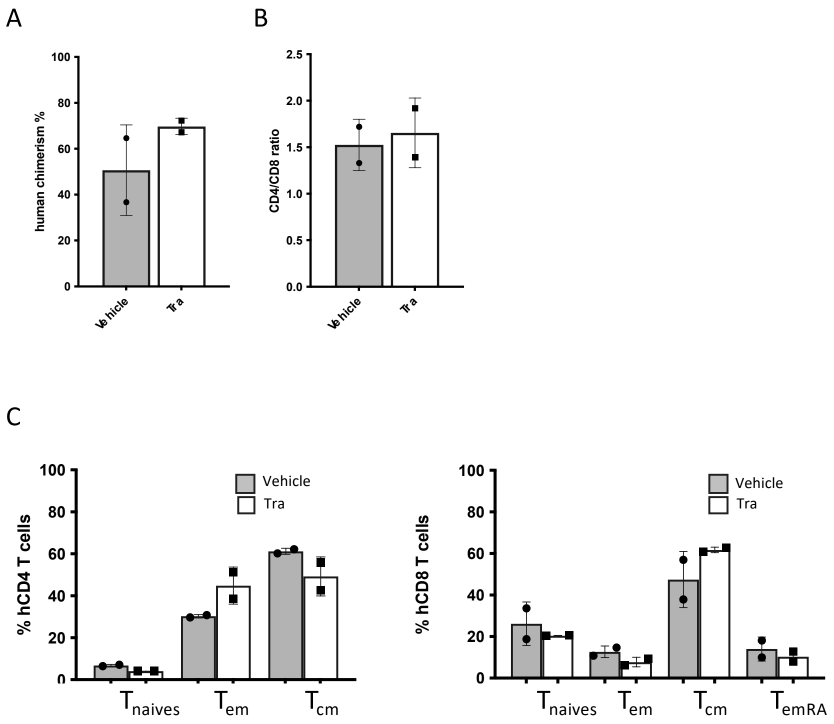

Supplementary Figure 7. Flow cytometry analysis of splenocytes harvested from vehicle and trametinib-treated animals (n=2 for each group) used for single-cell RNAseq experiment.
A) Bar plot showing human chimerism. (B) Bar plot showing the human CD4/CD8 ratio. (C) Bar plots showing the frequencies of hCD4 (right plot) and hCD8 (left plot) T cell populations analyzed by FACS as naive T cells, ($CD45RA^{+}CD27^{+}$), central memory ($CD45RA^{-}CD27^{+}$) and effector memory ($CD45RA^{-}CD27^{-}$) and TemRA ($CD45RA^{+}CD27^{-}$) T cells. Data are shown as means ± SD.

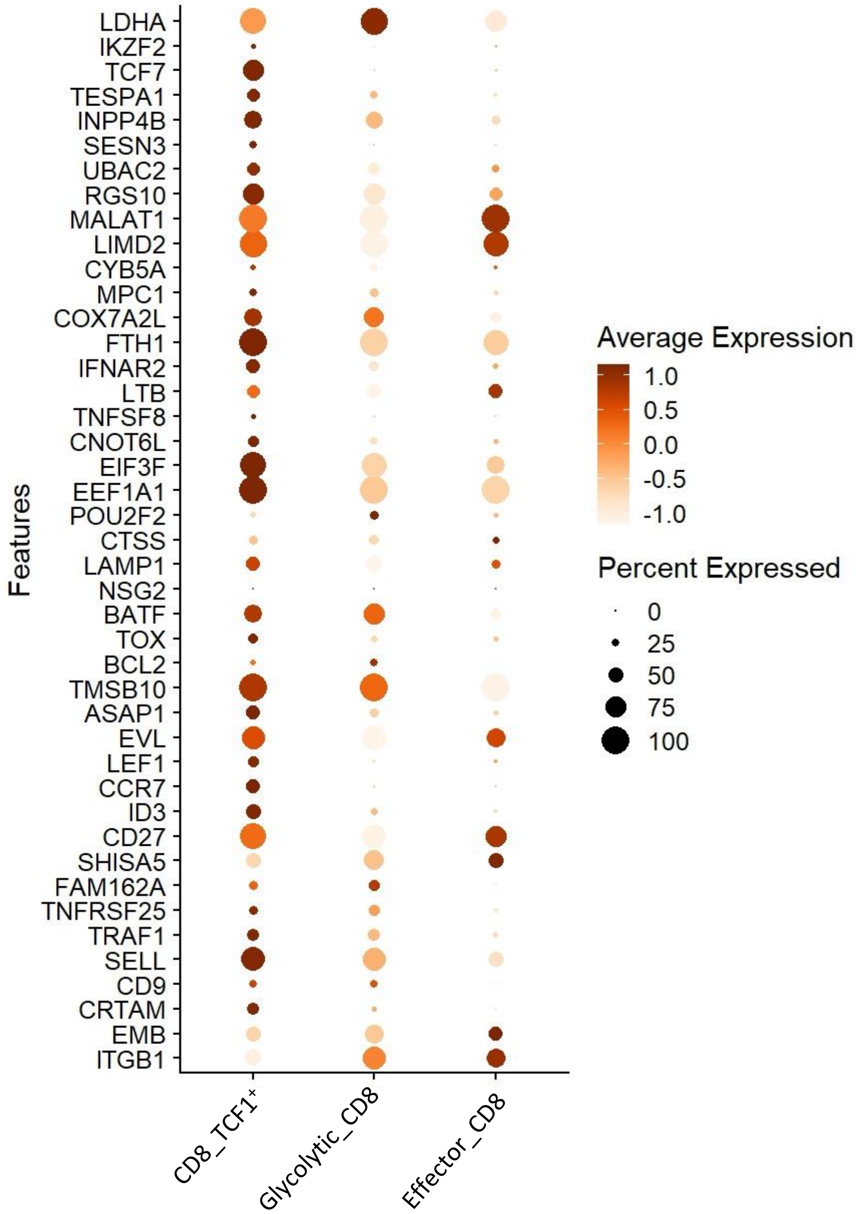

Supplementary Figure 8. Expression profiles of genes identified as markers base on differential expression analysis, across the CD8 clusters

Dot plot depicting the expression profiles (average normalized expression and percentage) of genes identified as markers base on differential expression analysis, across the three CD8 clusters.
